# Supplementary material for: Computer-aided discovery of novel SmDHODH inhibitors for schistosomiasis therapy: Ligand-based drug design, molecular docking, molecular dynamic simulations, drug-likeness, and ADMET studies
Source: PLoS Negl Trop Dis. 2024 Sep 12;18(9):e0012453. doi: 10.1371/journal.pntd.0012453 (PMC11392272; doi:10.1371/journal.pntd.0012453)
Supplement: S1 Table — (DOCX) [file pntd.0012453.s001.docx]

**Table S1**: Activities and structures of dataset compounds

| **S/N** | **Structures** | **pIC_50_** | **Predicted pIC_50_** | **Residual** | **Leverages** |
| --- | --- | --- | --- | --- | --- |
| 1 |  | 3.115 | 2.829 | 0.286 | 0.961 |
| 2 |  | 7.108 | 7.136 | -0.028 | 0.158 |
| 3 |  | 6 | 6.466 | -0.466 | 0.14 |
| 4 |  | 5.328 | 5.726 | -0.398 | 0.133 |
| 5 |  | 5.26 | 5.587 | -0.327 | 0.115 |
| 6 |  | 6.889 | 7.006 | -0.117 | 0.143 |
| 7 |  | 7.721 | 7.113 | 0.608 | 0.162 |
| 8 |  | 4.244 | 4.198 | 0.046 | 0.293 |
| 9 |  | 6.367 | 6.318 | 0.049 | 0.309 |
| 10 |  | 6.955 | 6.78 | 0.175 | 0.11 |
| 11 |  | 5.678 | 5.593 | 0.085 | 0.63 |
| 12 |  | 6.644 | 6.237 | 0.407 | 0.109 |
| 13 |  | 5.056 | 5.025 | 0.031 | 0.569 |
| 14 |  | 6.365 | 6.318 | 0.047 | 0.309 |
| 15 |  | 4.699 | 4.356 | 0.343 | 0.737 |
| 16 |  | 5.678 | 7.078 | -1.4 | 0.474 |
| 17 |  | 4.102 | 4.869 | -0.767 | 0.111 |
| 18 |  | 4.301 | 3.898 | 0.403 | 0.689 |
| 19 |  | 6.733 | 6.94 | -0.207 | 0.432 |
| 20 |  | 5.004 | 4.673 | 0.331 | 0.88 |
| 21 |  | 6.361 | 6.288 | 0.073 | 0.106 |
| 22 |  | 4.503 | 4.713 | -0.21 | 0.267 |
| 23 |  | 5.602 | 5.12 | 0.482 | 0.08 |
| 24 |  | 4.301 | 3.898 | 0.403 | 0.188 |
| 25 |  | 6.148 | 6.276 | -0.128 | 0.21 |
| 26 |  | 7.721 | 7.652 | 0.069 | 0.302 |
| 27 |  | 7.638 | 7.331 | 0.307 | 0.132 |
| 28 |  | 6.426 | 6.994 | -0.568 | 0.262 |
| 29 |  | 4.538 | 4.405 | 0.133 | 0.383 |
| 30 |  | 4.699 | 4.356 | 0.343 | 0.41 |
| 31 |  | 4.678 | 4.958 | -0.28 | 0.196 |

**Key**: training sets, test sets
