# Supplementary material for: Computer-aided discovery of novel SmDHODH inhibitors for schistosomiasis therapy: Ligand-based drug design, molecular docking, molecular dynamic simulations, drug-likeness, and ADMET studies
Source: PLoS Negl Trop Dis. 2024 Sep 12;18(9):e0012453. doi: 10.1371/journal.pntd.0012453 (PMC11392272; doi:10.1371/journal.pntd.0012453)
Supplement: S2 Table — (DOCX) [file pntd.0012453.s002.docx]

| **Table S2:** Interpretation and classes of the molecular descriptors within the developed model | | | | | |
| --- | --- | --- | --- | --- | --- |
| **S/N** | **Symbol** | **Description** | **Chemical Significance** | **Physical Significance** | **Class** |
| 1 | ***MATS3s*** | Moran autocorrelation - lag 3 / weighted by I-state. | Captures the spatial autocorrelation of the ionization state or polarizability of atoms over a distance of three bonds. | Reflects the distribution of electron density and polarizability within the molecule, important for understanding electronic interactions and reactivity. | 2D |
| 2 | ***VR2_Dzp*** | Normalized Randic-like eigenvector-based index from Barysz matrix / weighted by polarizabilities. | Involves the eigenvalues of a matrix constructed from atomic distances, weighted by atomic number (Z). | Captures the overall molecular size, shape, and atomic composition, reflecting geometric and electronic properties. | 2D |
| 3 | ***SpMin3_Bhm*** | Smallest absolute eigenvalue of Burden modified matrix - n 3 / weighted by relative mass. | Refers to the third smallest eigenvalue of the Burden matrix, weighted by atomic masses. | Indicates the contribution of atomic masses to the molecular structure, related to stability and mass distribution, affecting dynamics and reactivity. | 2D |
| 4 | ***SpMin4_Bhs*** | Smallest absolute eigenvalue of Burden modified matrix - n 4 / weighted by relative I-state. | Similar to SpMin3_Bhm, but considers the fourth smallest eigenvalue of the Burden matrix, weighted by ionization states. | Offers insight into the distribution of ionization states within the molecule, critical for understanding electronic properties related to ionization potential and reactivity. | 2D |
