# Supplementary figures and images for: Computer-aided discovery of novel SmDHODH inhibitors for schistosomiasis therapy: Ligand-based drug design, molecular docking, molecular dynamic simulations, drug-likeness, and ADMET studies
Source: PLoS Negl Trop Dis. 2024 Sep 12;18(9):e0012453. doi: 10.1371/journal.pntd.0012453 (PMC11392272; doi:10.1371/journal.pntd.0012453)

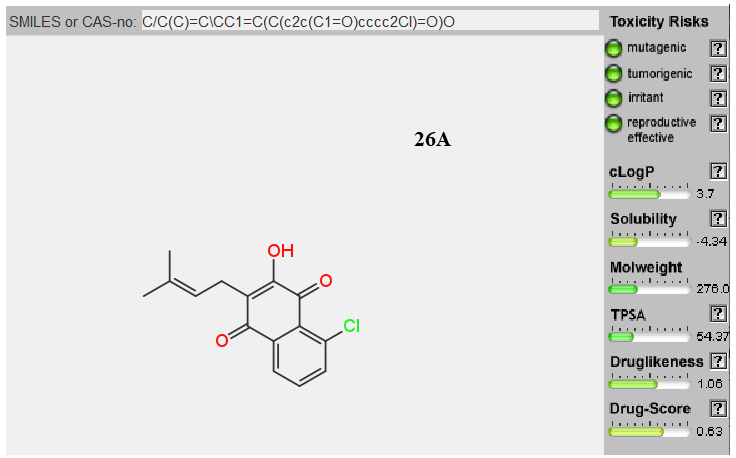


**Figure S1**: Profile of the designed compound (**26A**) showing its potential efficacy.

Supplement: S1 Fig — (DOCX) [file pntd.0012453.s003.docx]

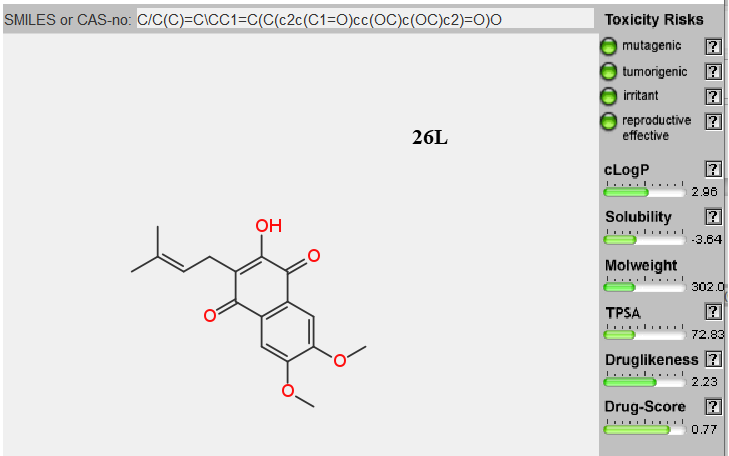


**Figure S2**: Profile of the designed compound (**26L**) showing its potential efficacy.

Supplement: S2 Fig — (DOCX) [file pntd.0012453.s004.docx]
