# Supplementary material for: Computer-aided discovery of novel SmDHODH inhibitors for schistosomiasis therapy: Ligand-based drug design, molecular docking, molecular dynamic simulations, drug-likeness, and ADMET studies
Source: PLoS Negl Trop Dis. 2024 Sep 12;18(9):e0012453. doi: 10.1371/journal.pntd.0012453 (PMC11392272; doi:10.1371/journal.pntd.0012453)
Supplement: S3 Fig — (DOCX) [file pntd.0012453.s005.docx]

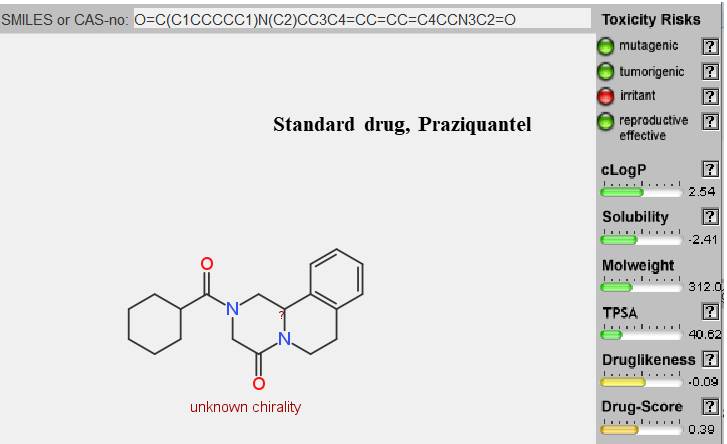


**Figure S3**: Profile of the Standard drug, Praziquantel (**PZQ**) showing its efficacy.

**Figure S3**: Profile of the Standard drug, Praziquantel (**PZQ**) showing its efficacy.
