# Supplementary material for: Computer-aided discovery of novel SmDHODH inhibitors for schistosomiasis therapy: Ligand-based drug design, molecular docking, molecular dynamic simulations, drug-likeness, and ADMET studies
Source: PLoS Negl Trop Dis. 2024 Sep 12;18(9):e0012453. doi: 10.1371/journal.pntd.0012453 (PMC11392272; doi:10.1371/journal.pntd.0012453)
Supplement: S4 Fig — 3-Dimentional 6UY4 interactions with designed compounds, (A) Complex with 26B; (B) Complex with 26C; (C) Complex with 26D; (D) Complex with 26E; (E) Complex with 26F; (F) Complex with 26G; (G) Complex with 26H; (H) Complex with 26I; (I) Complex with 26J; (J) Complex with 26K. (DOCX) [file pntd.0012453.s006.docx]

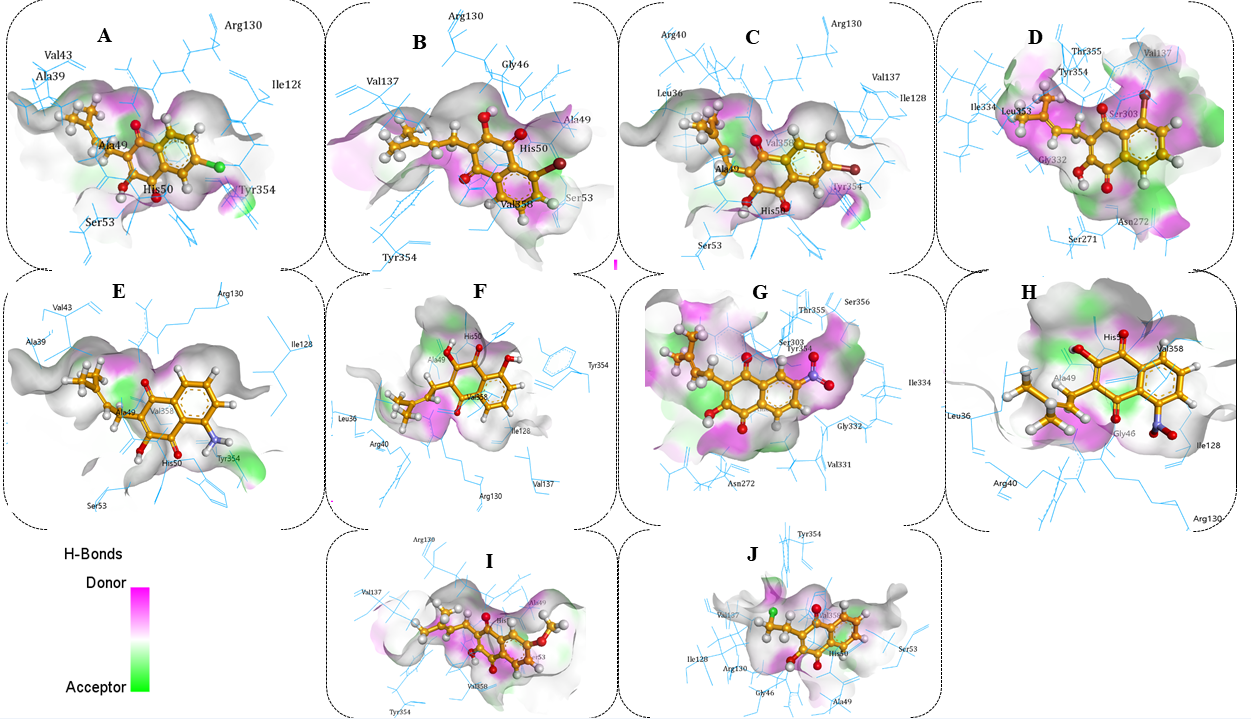


**Figure S4:** 3-Dimentional **6UY4** interactions with designed compounds, (**A**) Complex with **26B**; (**B)** Complex with **26C**; (**C)** Complex with **26D**; (**D)** Complex with **26E**; (**E)** Complex with **26F**; (**F)** Complex with **26G**; (**G)** Complex with **26H**; (**H)** Complex with **26I**; (**I)** Complex with **26J**; (**J)** Complex with **26K**.
