# Supplementary material for: Computer-aided discovery of novel SmDHODH inhibitors for schistosomiasis therapy: Ligand-based drug design, molecular docking, molecular dynamic simulations, drug-likeness, and ADMET studies
Source: PLoS Negl Trop Dis. 2024 Sep 12;18(9):e0012453. doi: 10.1371/journal.pntd.0012453 (PMC11392272; doi:10.1371/journal.pntd.0012453)
Supplement: Attachment — Submitted filename: Response to revieweres.docx [file pntd.0012453.s008.docx]

**Date:** 16.04. 2024

To

Editor-in-Chief

**PLOS NTD**

**Subject:** Submission of the revised manuscript

Dear Sir,

We have carried out essential revisions of our manuscript entitled “**Computer-aided discovery of novel SmDHODH inhibitors for Schistosomiasis therapy: Ligand-based drug design, Molecular docking, Molecular dynamic simulations, drug-likeness, and ADMET studies*.*”** suggested by the reviewer and would like to submit the 1^st^-revised version. We believe these comments/suggestions have significantly improved our manuscript. Please find enclosed the rebuttal letter where we have addressed all questions and comments to the reviewer, which are indicated as Yellow highlights in the manuscript. Please be noted that all authors consented and approved the revised version of the manuscript. We believe the updated version will be worthy of publishing in your journal.

Thanking you in advance for considering our work.

Yours Faithfully,

Abu Tayab Moin

E-mail: tayabmoin786@gmail.com

ORCID ID 0000-0002-2954-3215

**Author’s Responses to the Reviewers’ Comments**

**Reviewer #1**

**Comment 1**: Although the authors cited the ChEMBL ID that contains the compounds previously identified as inhibitors of SmDHODH, I believe that the corresponding publications that identified those compounds must be cited:

1 - Nonato, M. C., de Pádua, R. A., David, J. S., Reis, R. A., Tomaleri, G. P., Pereira, H. D. M., & Calil, F. A. (2019). Structural basis for the design of selective inhibitors for Schistosoma mansoni dihydroorotate dehydrogenase. Biochimie, 158, 180-190.

2 - Calil, F. A., David, J. S., Chiappetta, E. R., Fumagalli, F., Mello, R. B., Leite, F. H., ... & Nonato, M. C. (2019). Ligand-based design, synthesis and biochemical evaluation of potent and selective inhibitors of Schistosoma mansoni dihydroorotate dehydrogenase. European journal of medicinal chemistry, 167, 357-366.

**Response**: Thank you very much for this important observation. The primary reference sources that identified the compounds were cited in the revised manuscripts as the reviewer suggested.

**Comment 2**: The manuscript is well written and straightforward. Although I would like to see some of these newly identified compounds tested against the enzyme, the results are clear and brings novelty and significance, considering that very little is known regarding inhibition of the S. mansoni DHODH

**Response**: We deeply appreciate the reviewer's positive evaluation of the clarity and coherence of our manuscript. Your recognition of the novelty and significance of our research findings regarding the inhibition of Schistosoma mansoni DHODH is truly encouraging. We want to express our gratitude for your suggestion regarding experimental validation of the newly identified compounds against the enzyme. However, we must regretfully inform you that due to limited resources and lack of external funding support, we were unable to conduct experimental studies to test the inhibitory activity of these compounds against S. mansoni DHODH. Although we have underscored in the conclusion of our manuscript the importance of further synthesis and in vitro tests to confirm the predicted properties and assess their potential as anti-schistosomiasis agents. Despite these constraints, we are committed to exploring alternative avenues to validate our computational findings in the future.

**Reviewer #2**

**Comment 1**: The study aimed to identify potential inhibitors for the SmDHODH enzyme using computational approaches. Below are comments on the main aspects of the study:

Objectives and Hypothesis:

The study's objectives are reasonably clear, aiming to identify compounds that can act as inhibitors of the SmDHODH enzyme. However, there is confusion regarding the association between the action of these compounds as SmDHODH inhibitors and their subsequent efficacy against schistosomiasis. Clarifying this distinction is necessary for a clearer understanding of the objectives.

**Response**: Thank you for your valuable feedback on our study objectives. We acknowledge the importance of clarifying the association between the action of identified compounds as SmDHODH inhibitors and their subsequent efficacy against schistosomiasis. While our primary objective was to identify compounds capable of inhibiting the SmDHODH enzyme, we recognize the need to establish a clear link between enzyme inhibition and antischistosomal efficacy for a comprehensive understanding of our study objectives. We have revised the introduction part of our study to incorporate the link as suggested in the revised manuscript to ensure a clearer understanding of our research goals. Thank you for bringing this to our attention, and we appreciate your constructive input.

**Comment 2**: Study Design:

The study design, focused on computational approaches, seems appropriate to achieve the stated objectives. The methodology involved selecting compounds previously identified as inhibitors of the enzyme and utilizing them as a basis for computational studies, which included the development of QSAR models for designing potential enzyme ligands. The selected compounds were subjected to molecular docking and molecular dynamics studies to evaluate their interactions with the enzyme. Additionally, the compounds were evaluated for druglikeness parameters and prediction of ADMET properties.

However, it is crucial to recognize that the 31 compounds identified from CHEMBL lack sufficient chemical diversity for a broader study. Additionally, it's noted that the 31 selected compounds were not disclosed, which hinders transparency and reproducibility.

**Response**: Thank you for your thoughtful comments on our study. We appreciate your acknowledgment of the appropriateness of our computational approach in achieving the stated objectives. The methodology indeed focused on utilizing compounds previously identified as inhibitors of the enzyme, employing them as a basis for computational studies, including QSAR modeling for designing potential inhibitors, molecular docking, molecular dynamics studies, and evaluation of drug likeness parameters and ADMET properties. We acknowledge the limitation regarding the chemical diversity of the 31 compounds identified from CHEMBL, which is a valid concern for a broader study. Additionally, we would like to clarify that the dataset compounds were indeed provided in the supplementary materials file, ensuring transparency and reproducibility. We apologize for any confusion in our initial submission. We will consider your suggestions for including a more diverse chemical set in future studies to enhance the robustness and generalizability of our findings. Thank you for your valuable input, and we are committed to addressing these concerns in our future work.

**Comment 3**: Description of Population:

The target population, in the context of the computational study, consists of 31 compounds identified from CHEMBL. However, these compounds do not possess sufficient chemical diversity for a more comprehensive study. Unfortunately, the specific identities of these compounds were not provided, further complicating the assessment.

**Response**: Thank you for your thorough review and valuable feedback. We acknowledge the importance of a more diverse chemical set for a comprehensive study. In the revised manuscript, we have cited the original source of the compounds employed in our study, addressing the transparency regarding their identities. This inclusion should facilitate a clearer assessment and enhance the overall robustness of our research. Thank you for bringing these points to our attention, and we appreciate your constructive input.

**Comment 4**: Ethical and Regulatory Considerations:

No specific concerns about ethical or regulatory compliance in the context of the computational study were mentioned.

**Comment 5**: While the computational methodology employed in the study is adequate, the lack of chemical diversity among the identified compounds and the absence of experimental validation are significant limitations. Therefore, any conclusion at this point would be speculative. It is strongly recommended to conduct validation experiments using a more diverse set of compounds to strengthen the study's conclusions and enhance its scientific credibility.

**Response**: Thank you for your insightful review and valuable feedback. We appreciate your recognition of the adequacy of the computational methodology utilized in our study. However, we acknowledge the limitations posed by the lack of chemical diversity among the identified compounds and the absence of experimental validation, particularly considering our constraints as researchers from developing countries without external funding support. The experimental validations of the identified compounds is not within the scope of the current research. As such the title of the manuscript was re-casted in line with the research objectives as in the revised manuscript. Furthermore, we understand the importance of conducting validation experiments using a more diverse set of compounds to strengthen the credibility of our study's conclusions. While resource limitations may pose challenges, we are committed to exploring feasible approaches to address these limitations within our means. We will carefully consider your recommendation and aim to incorporate experimental validation in future research endeavors, prioritizing cost-effective strategies.

**Comment 6**: The manuscript presents a computationally relevant study aimed at identifying potential inhibitors for the SmDHODH enzyme. While the study lacks exploration of artificial intelligence techniques due to data limitations, it utilizes computational approaches effectively. However, the lack of chemical diversity among the identified compounds limits the scope of the study. Despite well-executed computational studies, the absence of experimental validation renders the obtained data speculative, which may not be suitable for publication in a journal like PLOS Neglected Tropical Diseases.

One notable concern is the conflation between SmDHODH inhibition and its consequent anti-parasitic action, which also requires validation. Although the computational studies were well-conducted, they should be complemented by experimental validation to strengthen the credibility of the findings.

Overall, the manuscript may overpromise compared to what it delivers. The title and author summary may exaggerate the significance of studying SmDHODH inhibition. Additionally, while the authors mention the problem of schistosomiasis in Africa, it's important to note that schistosomiasis is a global issue, and a broader perspective on the disease should be provided.

Furthermore, there is currently no clear evidence of resistance development to praziquantel, and the SmDHODH enzyme is not yet a validated target. However, identifying inhibitors for SmDHODH is an important step towards achieving this objective and warrants further investigation.

In conclusion, while the manuscript presents valuable computational insights, it falls short in terms of experimental validation and broader contextualization of the research. Addressing these shortcomings would significantly enhance the manuscript's scientific rigor and relevance.

**Response**: We appreciate the thorough review and constructive feedback provided by the reviewer. We acknowledge the limitations highlighted regarding our study and are committed to addressing them to enhance the scientific rigor and relevance of our manuscript.

Regarding the lack of exploration of artificial intelligence techniques, we recognize the potential benefits of leveraging such methods in drug discovery. However, due to data limitations, we focused on utilizing computational approaches effectively within the scope of our study.

As researchers from developing countries, we encounter challenges due to the lack of external funding and limited resources, which hinder our capacity to perform in vivo and in vitro studies as suggested. The financial constraints and absence of specialized facilities necessary for such experimental work present significant obstacles. Although we have underscored in the conclusion of our manuscript the importance of further synthesis and in vitro tests to confirm the predicted properties and assess their potential as anti-schistosomiasis agents. Nonetheless, our in silico drug design represents a pivotal step towards identifying potential treatments for schistosomiasis. It provides a cost-effective and efficient approach, particularly in resource-constrained settings, with the aim of offering valuable insights to the scientific community and addressing the health challenges confronted by populations in developing nations.

Furthermore, we acknowledge the reviewer's point regarding the broader perspective on schistosomiasis as a global issue and we have incorporated this in the introduction part of our revised manuscript.

Regarding the title and author summary, we have revise them to accurately reflect the scope and significance of our study, avoiding exaggeration. Additionally, we appreciate the clarification regarding resistance development to praziquantel and the validation status of SmDHODH as a target. We have revised the introduction part of our manuscript.

In conclusion, we thank the reviewer for their insightful comments. We value the opportunity to contribute meaningfully to the field of neglected tropical diseases and appreciate the guidance provided to enhance the scientific rigor of our manuscript.
