# Supplementary material for: Computer-aided discovery of novel SmDHODH inhibitors for schistosomiasis therapy: Ligand-based drug design, molecular docking, molecular dynamic simulations, drug-likeness, and ADMET studies
Source: PLoS Negl Trop Dis. 2024 Sep 12;18(9):e0012453. doi: 10.1371/journal.pntd.0012453 (PMC11392272; doi:10.1371/journal.pntd.0012453)
Supplement: Attachment — Submitted filename: reponse to reviewers (2nd revision).docx [file pntd.0012453.s009.docx]

**Date:** 15.07. 2024

To

Editor-in-Chief

**PLOS NTD**

**Subject:** Submission of the revised manuscript

Dear Sir,

We have carried out essential revisions of our manuscript entitled “**Computer-aided discovery of novel SmDHODH inhibitors for Schistosomiasis therapy: Ligand-based drug design, Molecular docking, Molecular dynamic simulations, drug-likeness, and ADMET studies*.*”** suggested by the reviewers and would like to submit the 2^nd^-revised version. We believe these comments/suggestions have significantly improved our manuscript. Please find enclosed the rebuttal letter where we have addressed all questions and comments to the reviewer, which are indicated as Yellow highlights in the manuscript. Please be noted that all authors consented and approved the revised version of the manuscript. We believe the updated version will be worthy of publishing in your journal.

Thanking you in advance for considering our work.

Yours Faithfully,

Abu Tayab Moin

E-mail: tayabmoin786@gmail.com

ORCID ID 0000-0002-2954-3215

**Author’s Responses to the reviewers’ Comments**

**Reviewer #1**: (No Response)

**Reviewer #3**: The article presents the findings of an in silico drug design, which intelligently integrates various complementary molecular modeling techniques. This integration facilitates the rational and accelerated development of drugs, as demonstrated by the results presented. However, the article could benefit from minor adjustments, particularly by carefully reviewing the text for repetitive passages. These repetitions may occur either in terms of expression (such as the consistent use of identical terms throughout the text, e.g., 'leverage') or in terms of redundant information.

**Response:** Thank you for your insightful feedback on our manuscript detailing the findings of our *in silico* drug design approach. We appreciate your positive assessment of the integration of diverse molecular modeling techniques to facilitate the rational and expedited development of drugs, as evidenced by the presented results. We acknowledge your suggestion to refine the manuscript by addressing potential repetitive passages. We have carefully review the text to ensure clarity and coherence, particularly by identifying and revising repeated terms and redundant information. This adjustment aims to enhance the overall readability and impact of the article.

Additionally, some crucial points require clarification:

**Comment 1**:    The authors mention the existence of resistance to praziquantel in the abstract. However, while this possibility has been observed in laboratory settings and there are instances of low responsiveness to treatment in African countries, to the best of my knowledge, this resistance is not conclusively proven. If the authors possess new information on this matter and can provide references, it would be important to disseminate it to the readership.

**Response**: Thank you for this important observation. The abstract has been modified in the revised manuscript.

**Comment 2:**   Statistical data lack accompanying errors (e.g., values of q2 and q2adj). Including this information is crucial.

**Response**: Thank you this important observation. In response to your comment regarding the lack of accompanying errors for statistical data, such as values of q2 and q2adj, we have now included a standard error value in the revised manuscript. This addition provides a measure of the precision in predicting the dependent variable, enhancing the reliability and interpretability of our findings.

**Comment 3:**   The authors introduce the constructed QSAR model and its validation, elucidating how independent variables influence pIC50. Nevertheless, no explanation has been given regarding the chemical and/or physical significance of the variables associated with the model's action. Therefore, it is imperative to explain the chemical and physical significance of parameters such as MATS3s, VR2_Dzp, SpMin3_Bhm, and SpMin4_Bhs.

**Response**: Thank you for your thorough review and insightful comments on our manuscript. We appreciate your feedback and the opportunity to clarify our work. In response to your comment regarding the chemical and physical significance of the variables associated with our QSAR model, we have provided detailed descriptions of these parameters in the supplementary material, specifically Table S2.

**Comment 4:**   Moreover, it appears to me that some of the parameters listed as important are indeed correlated (e.g.: MATS3s and SpMin3_Bhm; SpMin3_Bhm and SpMin4_Bhs). Please, explain those correlations coefficients.

**Response**: Thank you very much for this important observation. This has been reviewed and incorporated in the revised manuscript.

**Comment 5:**   Both the 31 compounds used as the basis for constructing and validating the QSAR model and the 12 newly proposed analogs lack their chemical structures in the provided information. It is crucial for these structures to be included in the main text rather than relegated to supplementary materials (especially the newly proposed ones).

**Response**: Thank you very much for this important observation. The molecular structures of the designed compounds is incorporated in the revised manuscript while that of the dataset compounds are in the revised supplementary material.

**Comment 6:**    In this context, Figure 4 suggests that these compounds are naphthoquinones. If indeed true, these compounds might carry potential toxicity. Solely relying on in silico-based toxicity predictions in this section of the article warrants reconsideration in light of existing literature information on these compounds.

Suggested Article’s DOI:

a.    10.3390/molecules190914902

b.    10.1080/01480545.2022.2104306

c.    10.1080/01480545.2022.2104306

d.    10.1080/01480545.2022.2104306

**Response**: Thank you for your insightful comments on our manuscript. We appreciate your feedback regarding Figure 4 and the potential toxicity of the compounds identified as naphthoquinones. We have carefully, studied the suggested literatures amongst others for the possible toxicity issue of naphthoquinone compounds. We have modified the discussion part of the results in the revised manuscript.

Moreover, we have utilized a reliable online tool to screen for drug likeness and pharmacokinetics parameters of the proposed derivatives, as detailed in our revised manuscript. As researchers from developing countries, we encounter challenges due to the lack of external funding and limited resources, which hinder our capacity to perform in vivo and in vitro studies. The financial constraints and absence of specialized facilities necessary for such experimental work present significant obstacles. Although we have underscored in the conclusion of our manuscript the importance of further synthesis and in vitro tests to confirm the predicted properties and assess their potential as anti-schistosomiasis agents. Nonetheless, our in silico drug design represents a pivotal step towards identifying potential treatments for schistosomiasis. It provides a cost-effective and efficient approach, particularly in resource-constrained settings, with the aim of offering valuable insights to the scientific community and addressing the health challenges confronted by populations in developing nations.

**Comment 7:** Finally, I'd like to make one last comment about the study: a 100 ns molecular dynamics simulation might be too brief to adequately demonstrate the stability of the analyzed complex. While I acknowledge that the authors may not have access to equipment for longer simulations, if feasible, extending the simulation to at least 300 ns would be advantageous.

**Response**: Thank you for highlighting the significance of molecular dynamics (MD) simulation in our study. Moreover, as researchers from developing countries, we encounter challenges due to the lack of external funding and limited resources, which hinder our capacity to perform the 300 ns MD simulations. The financial constraints and absence of specialized facilities necessary for such experimental work present significant obstacles. However, I will explore the possibility of incorporating such simulation in our future research.

--------------------
